# Diurnal Variation Has Effect on Differential Gene Expression Analysis in the Hippocampus of the Pilocarpine-Induced Model of Mesial Temporal Lobe Epilepsy

**DOI:** 10.1371/journal.pone.0141121

**Published:** 2015-10-16

**Authors:** Evelin Antonieli da Silva Santos, Thalita Ewellyn Batista Sales Marques, Heloísa de Carvalho Matos, João Pereira Leite, Norberto Garcia-Cairasco, Maria Luisa Paçó-Larson, Daniel Leite Góes Gitaí

**Affiliations:** 1 Department of Cellular and Molecular Biology, Institute of Biological Sciences and Health, Federal University of Alagoas, Maceio, Alagoas, Brazil; 2 Department of Neurology, Ribeirão Preto School of Medicine, University of São Paulo, Ribeirão Preto, São Paulo, Brazil; 3 Department of Physiology, Ribeirão Preto School of Medicine, University of São Paulo, Ribeirão Preto, São Paulo, Brazil; 4 Department of Cellular and Molecular Biology, Ribeirão Preto School of Medicine, University of São Paulo, Ribeirão Preto, São Paulo, Brazil; University of Modena and Reggio Emilia, ITALY

## Abstract

The molecular mechanisms underlying epileptogenesis have been widely investigated by differential gene expression approach, especially RT-qPCR methodology. However, controversial findings highlight the occurrence of unpredictable sources of variance in the experimental designs. Here, we investigated if diurnal rhythms of transcript’s levels may impact on differential gene expression analysis in hippocampus of rats with experimental epilepsy. For this, we have selected six core clock genes (*Per1*, *Per3*, *Bmal1*, *Clock*, *Cry1* and *Cry2*), whose rhythmic expression pattern in hippocampus had been previously reported. Initially, we identified *Tubb2a*/*Rplp1* and *Tubb2a*/*Ppia* as suitable normalizers for circadian studies in hippocampus of rats maintained to 12:12 hour light:dark (LD) cycle. Next, we confirmed the temporal profiling of *Per1*, *Per3*, *Bmal1*, *Cry1* and *Cry2* mRNA levels in the hippocampus of naive rats by both Acrophase and CircWave statistical tests for circadian analysis. Finally, we showed that temporal differences of sampling can change experimental results for *Per1*, *Per3*, *Bmal1*, *Cry1* and *Cry2*, but not for *Clock*, which was consistently decreased in rats with epilepsy in all comparison to the naive group. In conclusion, our study demonstrates it is mandatory to consider diurnal oscillations, in order to avoid erroneous conclusions in gene expression analysis in hippocampus of rats with epilepsy. Investigators, therefore, should be aware that genes with circadian expression could be out of phase in different animals of experimental and control groups. Moreover, our results indicate that a sub-expression of *Clock* may be involved in epileptogenicity, although the functional significance of this remains to be investigated.

## Introduction

Mesial temporal lobe epilepsy (MTLE) is a chronic disease characterized by spontaneous and recurrent seizures (SRS). The pathogenesis of MTLE involves structural and cellular reorganization of the hippocampal formation, including neuron loss, neurogenesis, gliosis, axonal damage or sprouting, dendritic plasticity, inflammation and reorganization of the extracellular matrix [1,2]. The molecular mechanisms underlying these processes have been widely investigated by differential gene expression approaches [3,4]. Combining different terms, such as ‘‘gene expression”, ‘‘pilocarpine”, ‘‘epilepsy” and ‘‘PCR”, we performed a PubMed search for articles published from January 1, 2005 to January 1, 2015 and got 57 available articles that evaluated gene expression changes by RT-PCR in the PILO-model. Surprisingly, when we confronted the data from independent studies carried out under similar experimental conditions, we found out that many genes showed controversial findings for the expression pattern during epileptogenic process, indicating unpredictable sources of variance in the experimental designs.

Real-time quantitative RT-PCR (RT-qPCR) is the dominant quantitative technique for analyzing RNA abundance because of its accuracy, sensitivity, specificity and speed [5–8]. However, in this type of analysis, the impact of experimental variations caused by technical (e.g., pipetting errors, reverse transcription efficiency, RNA quality and suitable normalizer) or biological (e.g., age, sex, tissue) factors can lead to inaccurate results and erroneous conclusions [9,10]. The recognition of these factors, therefore, is essential for a proper experimental design.

Diurnal rhythm has been described as a factor in many aspects of mammalian function, including the regulation of gene expression [11,12]. A master pacemaker located in the suprachiasmatic nucleus (SCN) of the hypothalamus drives these rhythms. The SCN integrates environmental information, based in both photic and non-photic stimuli, to synchronize circadian oscillations found throughout the body [13,14]. Hippocampus is a peripheral brain oscillator known to show robust circadian rhythms in morphological and physiological properties [15]. The molecular basis for circadian oscillation has been described as interlocked transcription-translation feedback loop, involving a set of clock genes, such as *Clock*, *Bmal1*, *Period* (Per)1, *Per2*, *Per3*, *Cryptochrome* (Cry) 1, *Cry2* and others [11,13,16–18]. These genes might control the circadian oscillation in genome-wide mRNA expression, which in turn regulate various biological processes. In fact, many studies using high throughput gene expression analysis have revealed that 9% to 30% of the transcriptome in cyanobacteria, *Arabidopsis*, *Drosophila* and mammals is under circadian control [19–23]. Despite this, most studies have underestimated the role of diurnal rhythms on transcript levels as an important source of variance in differential gene expression studies, especially those using RT-qPCR methodology.

Here, we evaluated the impact of diurnal variation on differential gene expression in hippocampus of rats with epilepsy. For this, we selected six clock genes (*Per1*, *Per3*, *Bmal1*, *Clock*, *Cry1* and *Cry2*), whose circadian or diurnal expression pattern in hippocampus have been reported [24–32]. As a first step, we investigated the most reliable reference genes for normalization of circadian studies in hippocampus of rats maintained to 12:12 hour light:dark (LD) cycle. Next, we analyzed, systematically, the temporal profiling of clock mRNA levels in the hippocampus of naive rats. Finally, we examined if temporal differences have an effect on results of differential expression analysis in the hippocampus of the Pilocarpine-induced epileptic rats. This model has been widely used for the study of the pathogenesis of temporal lobe epilepsy and to evaluate potential antiepileptogenic drugs [33].

## Materials and Methods

### Animals

Experiments were conducted on Wistar male rats (n = 49) from the main breeding stock of the Federal University of Alagoas, being 30 naive and 19 submitted to epilepsy induction protocol. All rats were 192–206 days-old and kept at 22± 2°C in groups of five per cage with free access to food and water. The animals were under a 12h light and 12h dark regimen, which was divided into 24-hour *Zeitgeber time* units (ZT), where ZT0 is when light is turned on (6 a.m.) and ZT12 when light is turned off (6 p.m.).The rats were divided into naive and epileptic groups. Naive Rats were euthanized every 4 hours during a 24h period (five animals per time point) at the ZT 0, 4, 8, 12, 16 and 20. Epileptic rats were euthanized at ZT8 and ZT12, five animal per time point. Since the choice of any one of the sacrificial points for epileptic rats would allow answering the purposes of this investigation, there was not a specific criterion for choosing ZT08 and ZT12, except for greater convenience of time of the day. All the animals were euthanized by decapitation using a guillotine within 20min of the each *Zeitgeber times*.

All animal experiments were performed in accordance with a protocol approved by the Research Ethics Committee of the Federal University of Alagoas (Permit number: 02/2012) and were consistent with the International guidelines of the ethical use of animals, such as those from the Society for Neuroscience. The research staff monitored rats at least twice every week for signs of illness or impairment by observing the general body condition, respiration rate, dehydration, posture, immobility, social interaction and response to manipulation. For the animals submitted to *status epilepticus* (SE), monitoring the health was carried out for 8–10 hours/day until the complete post-ictal recovery (lasted up to 2 days after SE). During this period, all efforts were made to minimize the suffering of the animals by electrolyte and nutrient replacement (i.p saline 0.9% and glucose 5%; and by feeding animals with pasty food). No animals presented clinical/behavior signal of pain or unexpected distress used as humane endpoint criteria for euthanasia. All efforts were made to reduce the number of animals used and to avoid any unnecessary suffering.

### Pilocarpine-induced model of mesial temporal lobe epilepsy

Animals were injected intra-peritoneally (i.p) with scopolamine butyl-bromide (1mg/kg) in order to reduce peripheral cholinergic effects, followed 30 min by PILO in a dose of 300mg/kg. All animals that had SE were rescued with diazepam (5mg/kg; i.p), 90 min after SE establishment. Out of 19 PILO-injected rats, 9 died during the SE, and 10 survived to SE. Indeed, the SE is associated with high mortality rates for male Wistar rats treated with 300–400 mg/kg of PILO, that is able to lead to the chronic phase of epilepsy [33–36]

From the third day after SE, animals were individually placed in acrylic cages and their behavior was recorded on videotapes for up to 6 hours per day, during 65 days. All the videos were analyzed by two independent observers and the seizure severity was classified according to Racine scale [37]. Only animals with two or more limbic seizures equal or greater than 3 values in the Racine´s scale were included in the molecular analysis (5 and 4 rats for ZT8 and ZT12 groups, respectively). After 12 weeks from PILO-induced SE, the epileptic animals were euthanized to tissue collection.

### RNA extraction and reverse transcription

Hippocampi were rapidly isolated on an ice-chilled plate and immediately frozen and stored in liquid nitrogen until RNA extraction. Total RNA was purified using Trizol reagent (Invitrogen, CA, USA), following the manufacturers protocol. Total RNA was treated with DNase I (Ambion, TX, USA) for 30 min in order to avoid amplification of genomic DNA.

Total RNA (1μg) from the left hippocampus of each rat was reverse transcribed to single stranded cDNA using the High-Capacity cDNA Reverse Transcription Kit (Applied Biosystems, Foster City, CA) according to manufacturer’s instructions. Once reverse-transcription was complete, samples were diluted (10X) in TE (Tris 10mM, pH 7,4; EDTA 0,1mM, pH 8,0) and stored at –80°C until further analysis.

### Real time PCR

Real-time analysis was carried out on StepOnePlus™ Real Time PCR systems (Applied Biosystem, CA, and USA). Amplifications were performed in 12.5μL volume reactions containing cDNA (2.5μL), 0.2–0.6μM each of specific forward (F) and reverse (R) primers, and 6μl Power Syber® Green PCR Master Mix (Applied Biosystem, CA, USA). For clock genes, all primer sequences and characteristics are listed in Table 1, whereas for candidate reference genes, see Marques et al. [38]. The amplification protocol used was as follows: initial 10min denaturation and 40 cycles of 95°C for 15s and 60°C for 1min. To ensure specificity of the PCR amplicon, a temperature controlled melting curve analysis was performed as a last step of the PCR reaction. As expected, each melting curve revealed a single peak, corresponding to the desired specific amplification product, with exception for *Per1*, which had the specificity of amplicon confirmed on 8% polyacrylamide gels. For all genes, the absence of contamination was confirmed by PCR amplification using a no template control (NTC), with water in place of cDNA, on every plate. Each assay was performed in triplicate and the mean values were used for further analysis. To estimate the efficiencies of amplification, a standard curve was generated for each primer pair based on 5 points of serial dilution of pooled cDNA (1:20; 1:40; 1:80; 1:160 and 1:320). All calibration curves exhibited a real-time PCR efficiency ranging 90 to 110% (Table 1).

**Table 1:** Primer sequences and amplification summary.

Gene*	Reference	*5'-3' sequence*	Amplicon lenght (pb)	PCR efficiency (%)
*Clock*	AB019258.1	F-CTTCAGTTCAGCAGCCAGC	125	109,00
		R-GCTCTGTTGTAGTGGAAAGGCA		
*Cry1*	NM_198750.2	F-CAGTTGGGAAGAAGGGATGAAG	60	99,78
		R-ATGCTCCAGTCGGCGTCAA		
*Per1*	NM_001034125.1	F-GCAGAAACAACAGCCACGGT	115	103,93
		R-GTCCACACAAGCCGTTACATCG		
*Per3*	NM_023978.2	F-CCACAGCATCAGTACAGCAAG	142	90,22
		R-GCTCTGTCTCTCTGTCTATCCT		
*Bma1l*	NM_024362.2	F-CCGTGGACCAAGGAAGTAGA	97	97,66
		R-CTGTGAGCTGTGGGAAGGTT		
*Cry2*	NM_133405.2	F-ATTGAGCGGATGAAGCAGAT	103	98,63
		R-CCACAGGGTGACTGAGGTCT		

*Clock, Circadian locomotor output cycle kaput; Cry1-2, Cryptochrome1-2; Per1-3, Period 1–3; Bmal1, Brain and muscle Arnt-like protein-1.

All the target gene expression was normalized to the most stable combination (*Tubb2a/Rplp1*), as determined by geNorm and NormFinder analyses. Relative amounts of transcripts were calculated using the 2^-ΔΔCt^ method [39]. Values were expressed in quantities relative to the calibrator, which was run on each PCR plate through the entire experiment.

### Selection of reference gene

Eight commonly used reference genes beta-actin (*Actb*), beta-2-microglobulin (*B2m*), glyceraldehyde-3-phospate dehydrogenase (*Gapdh*), beta-glucuronidase (*Gusb)*, beta-tubulin (*Tubb2a*), peptidylprolyl isomerase A (*Ppia*), ribosomal protein, large, P1 (*Rplp1*) and polymerase (RNA) I polypeptide A (*Polr1a*) were selected and their expression measured in the hippocampus of Wistar rats at different ZT of a 12:12 light-dark cycle.

Initially, we assessed the stability of candidate reference genes using two commonly and publicly available programs named geNorm and NormFinder. For this, Ct values were converted into relative quantities via the delta-Ct method using the sample with the lowest Ct as calibrator, in accordance with the method [39]. GeNorm calculates the stability of selected reference genes according to the similarity of their expression profile by pair-wise comparison and then calculates M value, a gene expression stability factor, where a lower M value indicates higher stability of the reference gene. The program also estimates the pairwise variation between two sequential calculations of normalization factors (NF) including an increasing number of genes. This defines the minimal number of genes required to calculate a robust normalization factor. GeNorm defines a pairwise variation of 0.15 as the cutoff value, below which the inclusion of an additional reference gene is unnecessary. NormFinder uses an ANOVA-based model to estimate intra- and inter-group variation, and combines these estimates to provide a direct measure of variation in expression levels for each gene.

In order to validate the best reference genes, we evaluated *Per1* expression profile in hippocampus of rats at different *Zeitgeber* times after normalization with the most stable combination reference genes.

### Statistical analysis

Statistics were performed using GraphPad Prism 5.00 (GraphPad Software, Inc. San Diego, CA, USA). Unpaired Student’s t-test was used for comparison of gene expression results between epileptic (ZT8) and each ZT of naive group, separately. Mean differences were considered statistically significant when P<0.05. For analysis of diurnal patterns of clock genes expression, both Acrophase (http://www.circadian.org/softwar.html) and CircWave (http://www.euclock.org/results/item/circ-wave.html) softwares were used. The CircWave employs a forward linear harmonic regression to calculate the profile of the wave with a 24h period. A 24-hour rhythm was confirmed if the null amplitude hypothesis was rejected from an F test producing a significant value (p<0.05). The Acrophase uses the cosinor method that fits one (or several) cosine curve(s) by least squares to the data, yielding estimates for the amplitude (half the difference between the minimum and maximum of the fitted cosine function), mesor (middle value of the fitted cosine curve representing the rhythm adjusted mean) and acrophase (Φ, time of peak value of the fitted cosine function). Based on the residual sum of squares, a P value was derived for the zero-amplitude (no-rhythm) test and for the computation of confidence intervals of 95% for the parameters. A p<0.05 was taken as indicative of the presence of a rhythm with the 24h (anticipated) period.

## Results

### Selection of reference genes for circadian studies and temporal profiling of clock transcript levels in hippocampus of naive rats

**Fig 1:**
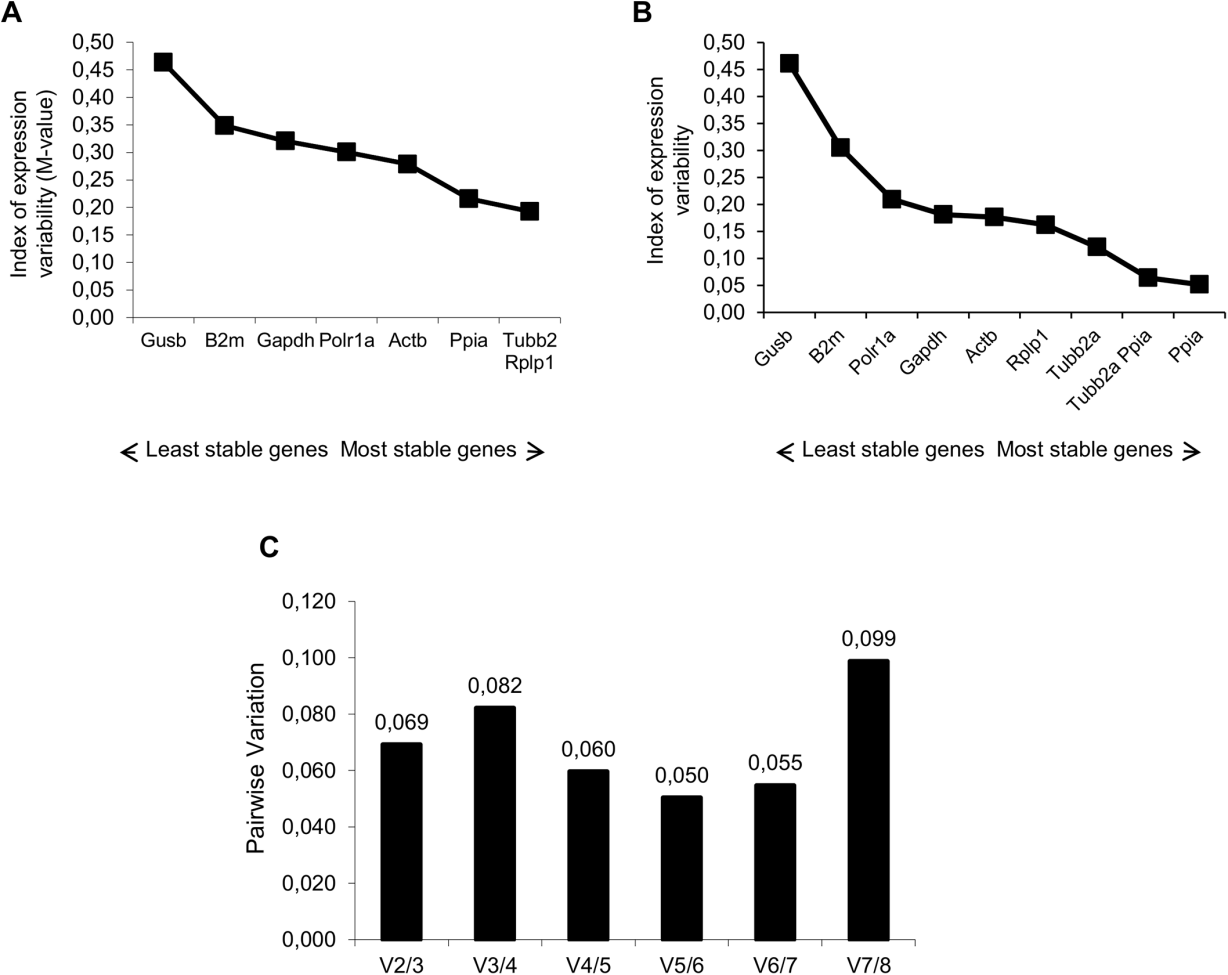
Selection of the most suitable reference genes for circadian analysis in the hippocampus of rats. Expression stability measurements for the 8 reference genes calculated by geNorm (A) and NormFinder (B). The x-axis from left to right indicates the ranking of the genes according to their expression stability; lower values indicate higher expression stability. C) Determination of the optimal number of reference genes for normalization by geNorm. The Software calculates the normalization factor from at least two genes at which the variable V defines the pair-wise variation between two sequential normalization factors.

**Fig 2:**
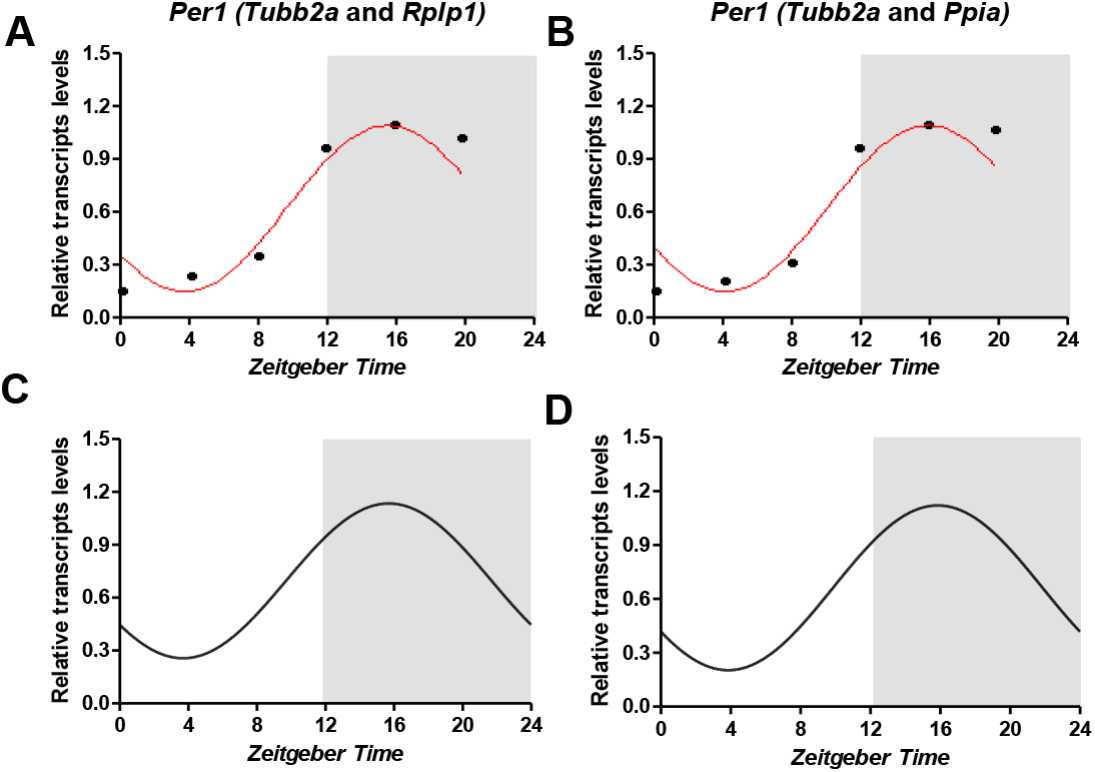
Temporal profile of *Per1* transcripts in the hippocampus based in two different normalization strategies. RT-qPCR data were normalized by best combination of genes derived by geNorm (A,C) and NormFinder (B,D) analysis (mean, n = 5). Statistical test for circadian analysis by Acrophase (A, B) and CircWave (C, D). Data are presented as mean (n = 5 rats/time point).

Our first aim was to identify genes that could be used as normalizers for RT-qPCR analysis in hippocampus of Wistar rats throughout a 12:12 light-dark cycle. We evaluated expression stability of the candidate genes in hippocampus samples harvest at different ZTs, using geNorm and Normfinder softwares. The average expression stability values (M values) of the reference genes in all tested samples are displayed in Fig 1A. All the genes presented high expression stability, with the M values varying between 0.21 (*Ppia*) and 0.46 (*Gusb*). The pairwise variation V2/3 was 0.069 (Fig 1C); thus, the *Tubb2a*/*Rplp1* genes were indicated as the optimal pair to provide normalization of gene expression at the different photoperiods tested. Results of NormFinder analysis are shown in Fig 1B. Also, *Ppia* and *Gusb* appeared, as the most and the least stable genes (stability value of 0.052 and 0.46), respectively. The best combination of reference genes indicated was *Tubb2a*/*Ppia*. These data sets are comparable with those obtained using geNorm, with slight differences in the ranking order of the most stable genes and of the best pair combination. In order to validate the results obtained, we conducted a relative expression analysis of the *Per1* gene. We used the recommended combination of genes from both geNorm (*Tubb2a*/*Rplp1*) and NormFinder (*Tubb2a*/*Ppia*) as internal controls. The statistical analysis of *Per1* diurnal expression was performed by Acrophase and CircWave softwares. We observed that with both normalization procedures, a *Per1* transcript levels oscillate in a rhythmic pattern, peaking at approximately ZT16 and with amplitude nearly of 0.47 (Fig 2).

**Fig 3:**
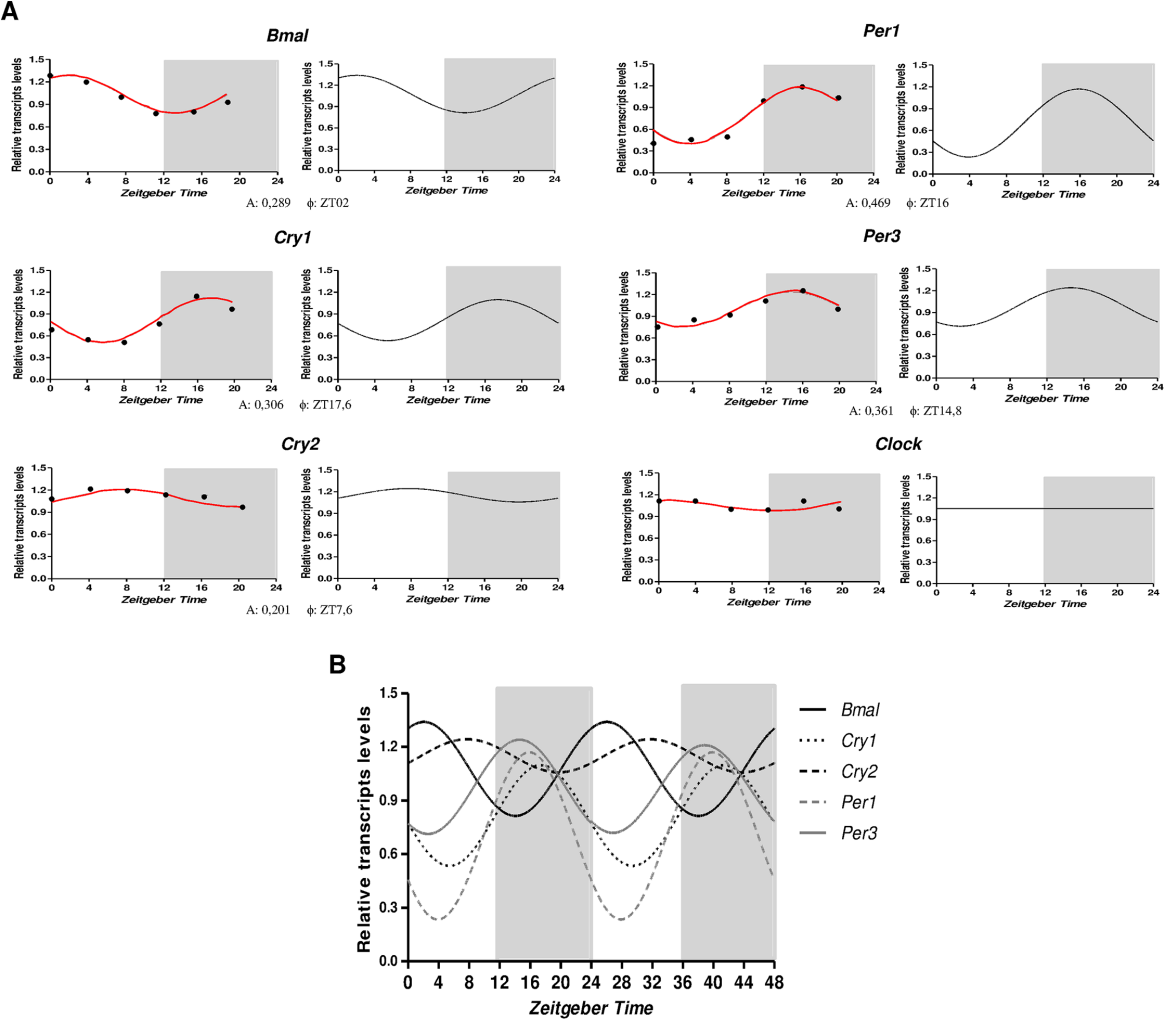
Temporal expression of the core clock transcripts in the hippocampus of rats. A) Relative amounts of transcripts at different ZT after normalization to *Tubb2a*/*Rplp1*. Data are presented as mean (n = 5 rats/ZT). Statistical test for circandian analysis by Acrophase (left) and CirWawe (right). B) Overlap of cosine fitting curves illustrating the phase relation of clock transcripts. For clarity reasons, data are doubleblotted against *Zeitgeber time* (ZT).

Following, we used T*ubb2a*/*Rplp1* as normalizers to evaluate, systematically, the temporal profiling of *Bmal1*, *Per1*, *Per3*, *Cry1*, *Cry2* and *Clock* mRNA levels in the hippocampus of rats sacrificed every 4 hours during a 24-h period. Fig 3 illustrate the temporal organization and phase relationship of the clock genes analyzed. Using both Acrophase and CircWave softwares, we observed that with exception of *Clock*, all genes showed a rhythmic pattern of expression, being *Per1* and *Cry2* those with the highest (0.469) and lowest (0.201) amplitude, respectively (Fig 3A). The comparison of the rhythms of each gene showed that *Bmal1* (Φ = ZT2) is in antiphase with *Per1* (Φ = ZT16), *Per3* (Φ = ZT14.8) and *Cry1* (Φ = ZT17.6), whereas *Bmal1* peak is approximately 5 hours before acrophase of *Cry2* (Φ = ZT7.6) (Fig 3B).

### Effects of diurnal variation on differential gene expression analysis in hippocampus of epileptic rats

**Fig 4:**
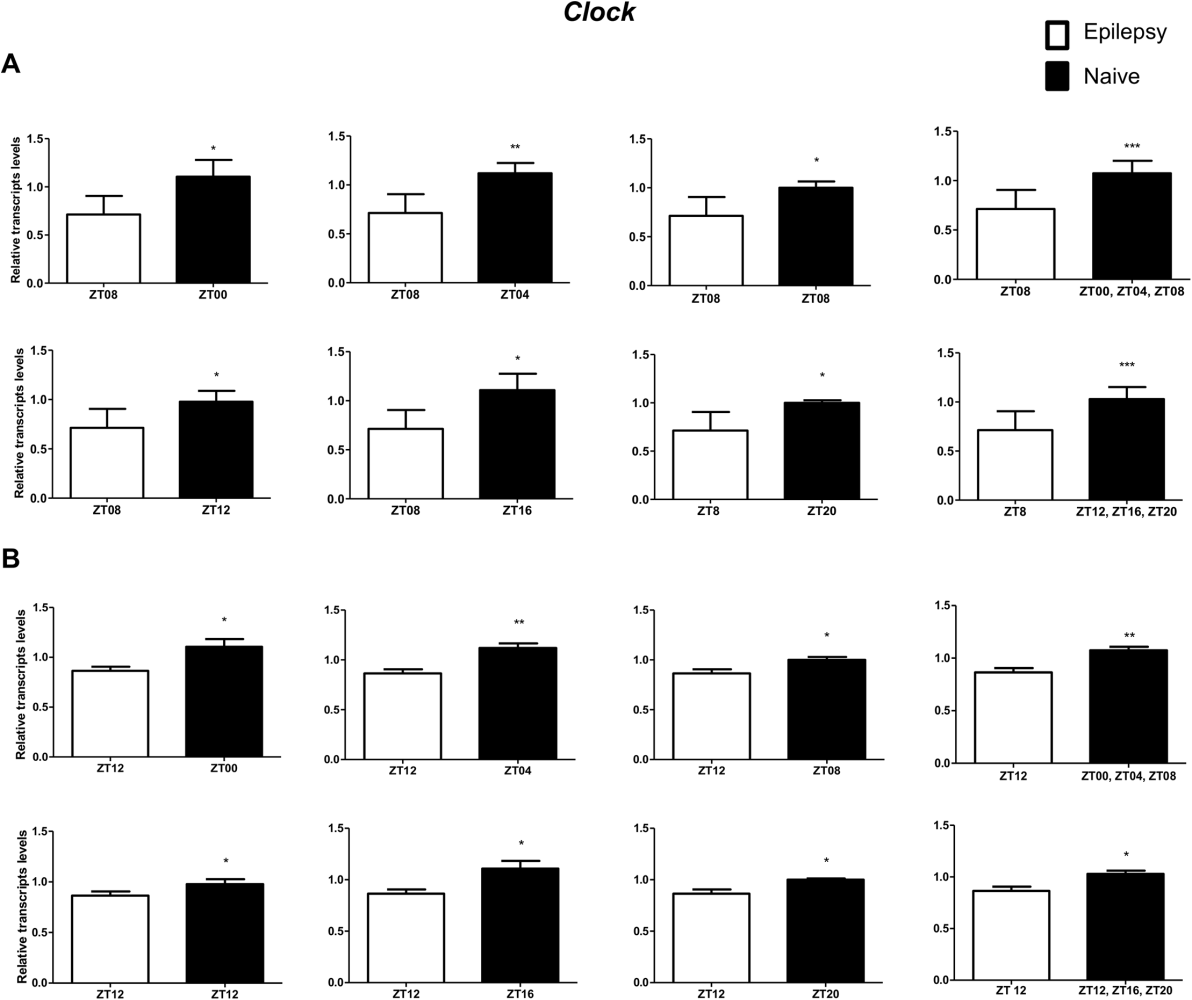
Impact of diurnal variation on *Clock* expression analysis in hippocampus of epileptic rats. Relative amounts of *Clock* transcripts in epileptic rats ZT08 (A) and ZT12 (B) after normalization to *Tubb2a*/*Rplp1*. Significant differences were evaluated using Unpaired Student’s t-test comparing results between epileptic and each ZT of naive group. *p<0.05, **p<0.01 and ***p<0.001. Data are presented as mean+SEM (n = 4 rats in each epileptic group and 5 rats/time point in naive).

**Fig 5:**
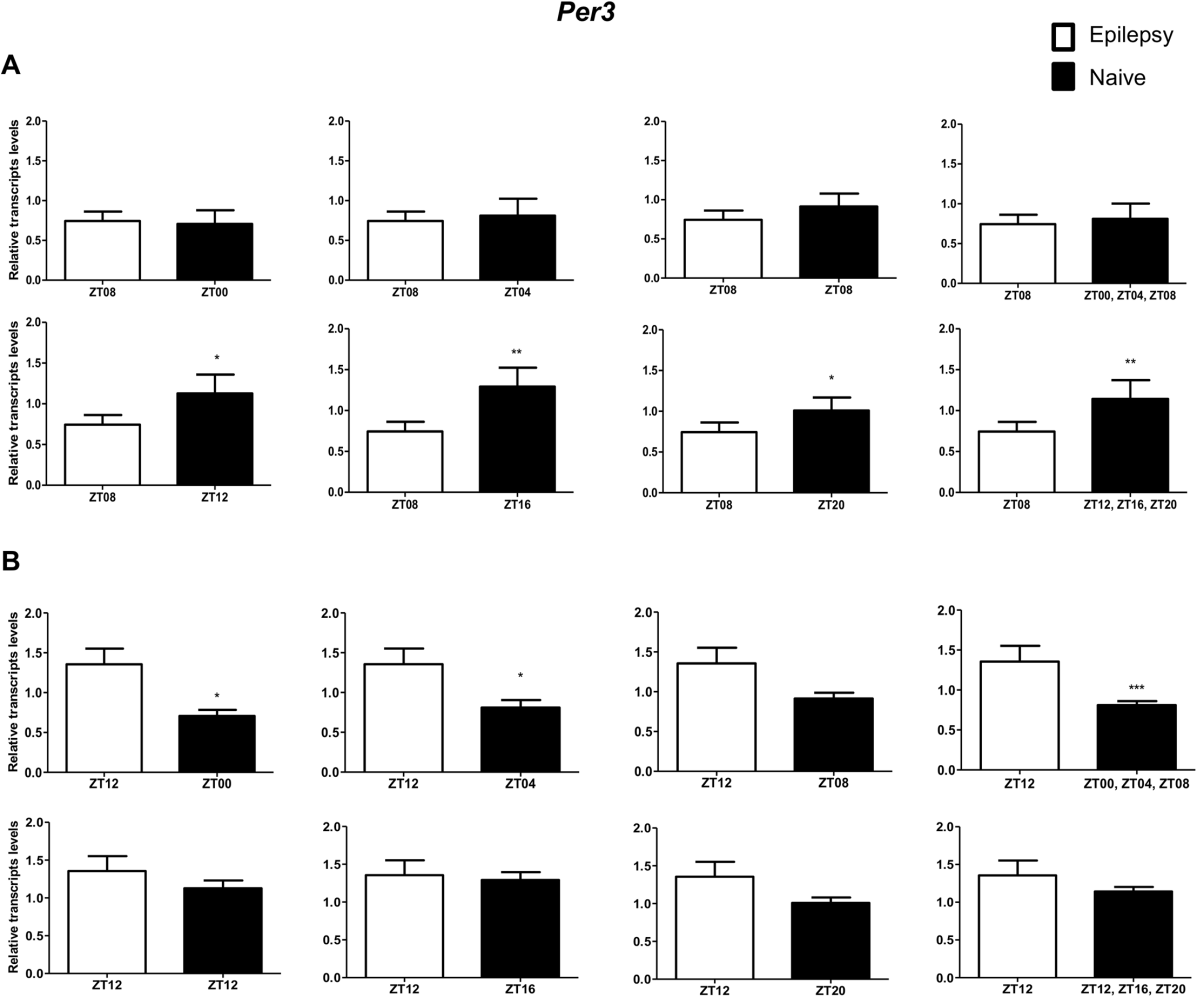
Impact of diurnal variation on *Per3* expression analysis in hippocampus of epileptic rats. Relative amounts of *Per3*, transcripts in epileptic rats ZT08 (A) and ZT12 (B) after normalization to *Tubb2a*/*Rplp1*. Significant differences were evaluated using Unpaired Student’s t-test comparing results between epileptic and each ZT of naive group. *p<0.05, **p<0.01 and ***p<0.001. Data are presented as mean+SEM (n = 5 (ZT8) and 4 (ZT12) rats in epileptic group and n = 5 rats/time point in naive).

**Fig 6:**
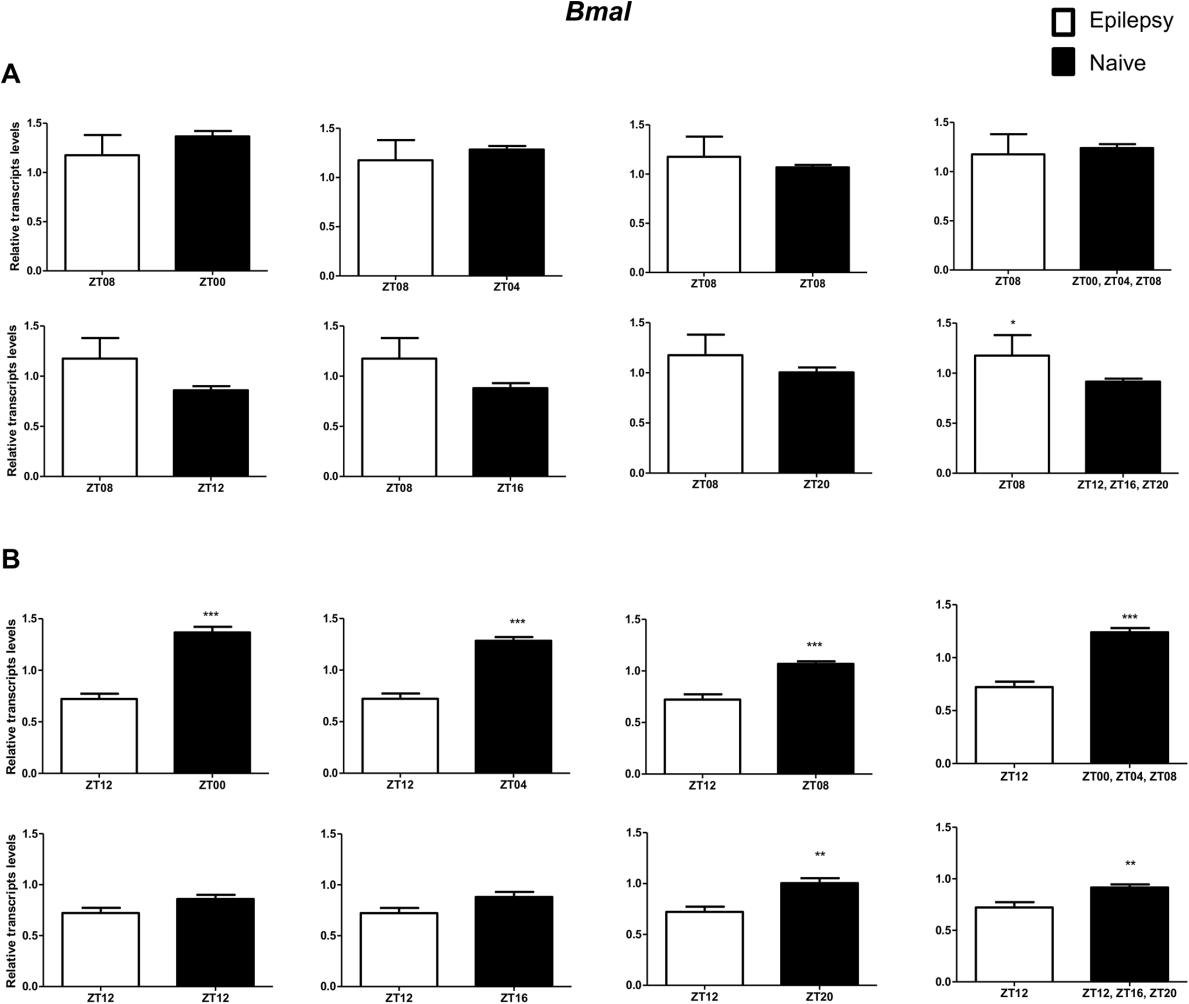
Impact of diurnal variation on *Bmal* expression analysis in hippocampus of epileptic rats. Relative amounts of *Bmal*, transcripts in epileptic rats ZT08 (A) and ZT12 (B) after normalization to *Tubb2a*/*Rplp1*. Significant differences were evaluated using Unpaired Student’s t-test comparing results between epileptic and each ZT of naive group. *p<0.05, **p<0.01 and ***p<0.001. Data are presented as mean+SEM (n = 5 (ZT8) and 4 (ZT12) rats in epileptic group and n = 5 rats/time point in naive).

**Fig 7:**
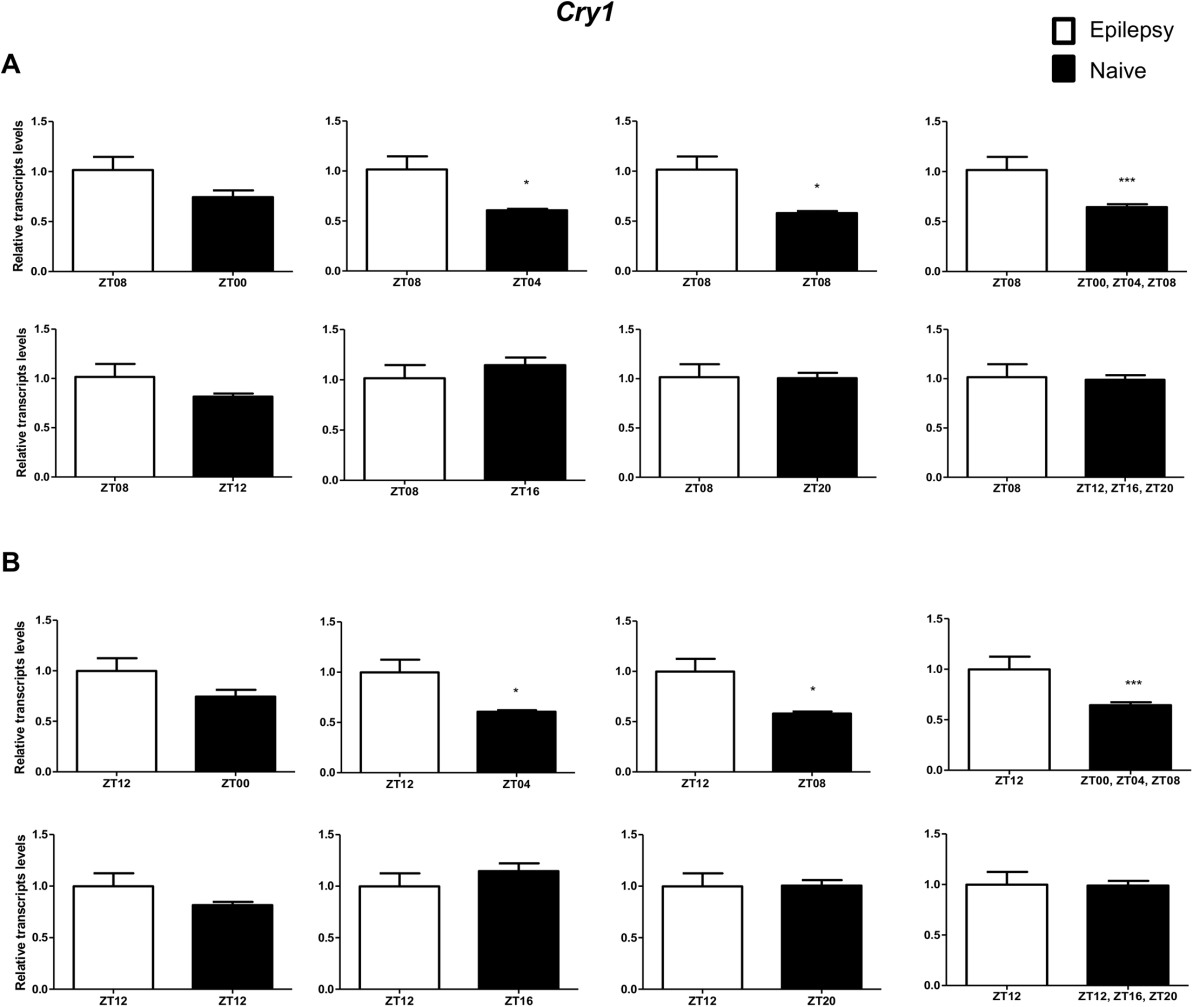
Impact of diurnal variation on *Cry1* expression analysis in hippocampus of epileptic rats. Relative amounts of *Cry1*, transcripts in epileptic rats ZT08 (A) and ZT12 (B) after normalization to *Tubb2a*/*Rplp1*. Significant differences were evaluated using Unpaired Student’s t-test comparing results between epileptic and each ZT of naive group. *p<0.05, **p<0.01 and ***p<0.001. Data are presented as mean+SEM (n = 5 (ZT8) and 4 (ZT12) rats in epileptic group and n = 5 rats/time point in naive).

**Fig 8:**
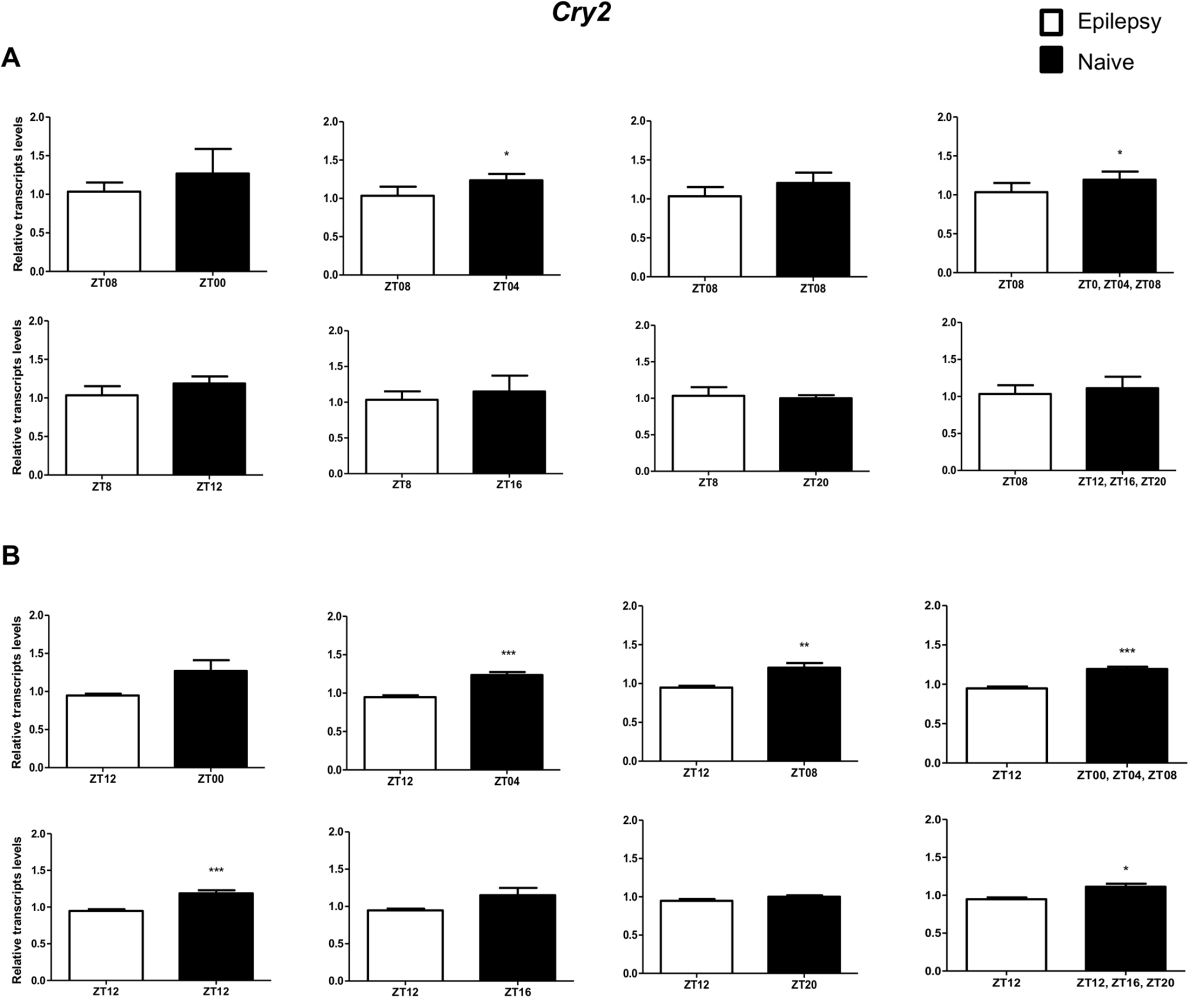
Impact of diurnal variation on *Cry2* expression analysis in hippocampus of epileptic rats. Relative amounts of *Cry2*, transcripts in epileptic rats ZT08 (A) and ZT12 (B) after normalization to *Tubb2a*/*Rplp1*. Significant differences were evaluated using Unpaired Student’s t-test comparing results between epileptic and each ZT of naive group. *p<0.05, **p<0.01 and ***p<0.001. Data are presented as mean+SEM (n = 5 (ZT8) and 4 (ZT12) rats in epileptic group and n = 5 rats/time point in naive).

**Fig 9:**
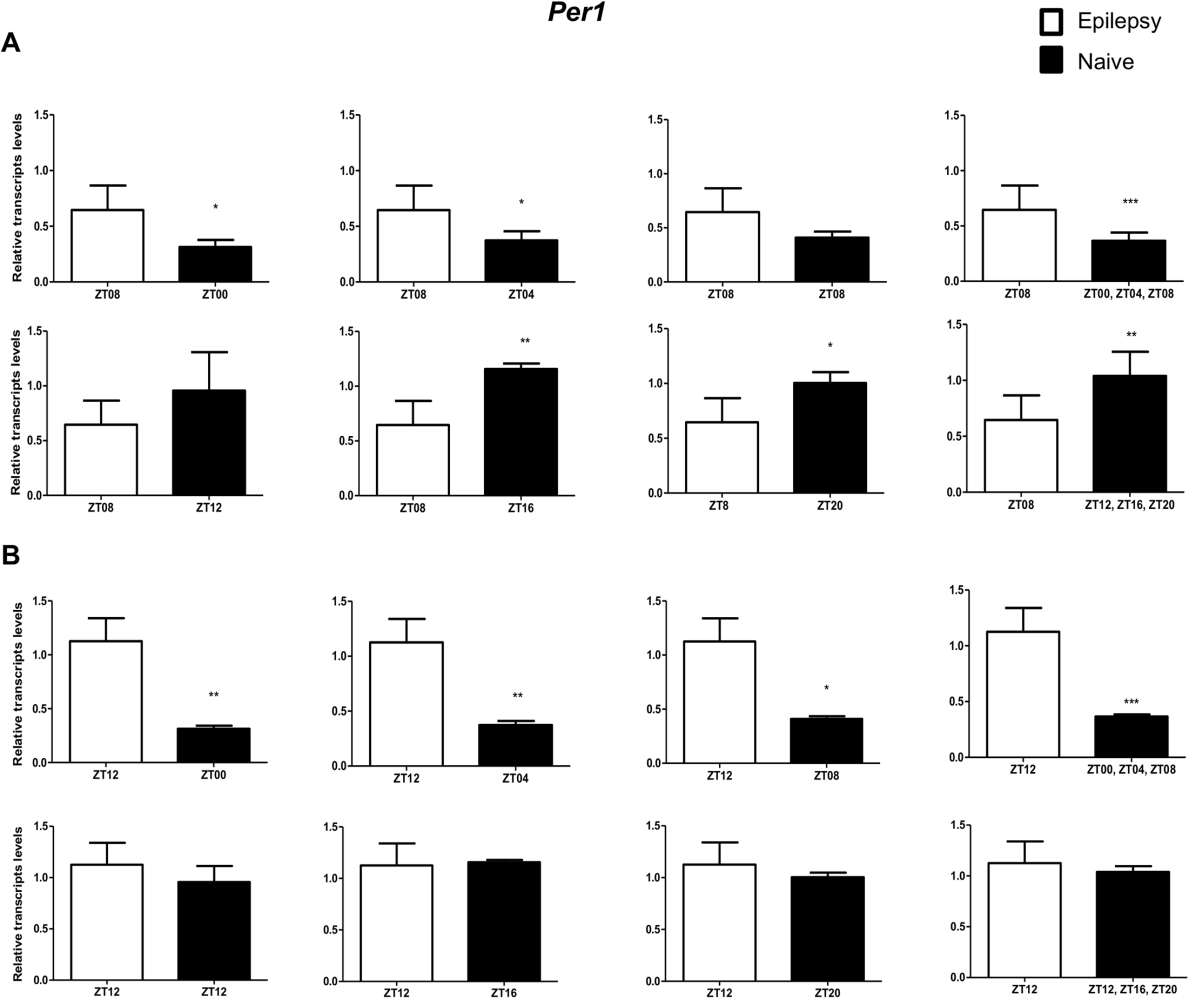
Impact of diurnal variation on *Per1* expression analysis in hippocampus of epileptic rats. Relative amounts of *Per1*, transcripts in epileptic rats ZT08 (A) and ZT12 (B) after normalization to *Tubb2a*/*Rplp1*. Significant differences were evaluated using Unpaired Student’s t-test comparing results between epileptic and each ZT of naive group. *p<0.05, **p<0.01 and ***p<0.001. Data are presented as mean+SEM (n = 4 rats in each epileptic group and 5 rats/time point in naive).

To investigate if the diurnal expression could be a source of variability on differential gene expression analysis in hippocampus of epileptic rats, we compared expression levels of the clock genes between epileptic rats sacrificed at ZT8 or ZT12 with naive rats sacrificed at different *Zeitgeber times*. In relation to ZT8 epileptic rats, *clock* transcripts were significantly decreased in the hippocampus of epileptic rats in all comparisons with the naive group (Fig 4A). However, the *Per3* was decreased only when compared with naive rats correspondent to ZT points of the dark phase (Fig 5A). *Bmal1* transcripts were significantly increased in epileptic rats only when compared with naive rats relative to the combination of dark phase ZTs (Fig 6A). Significant differences in *Cry1* (increase) and *Cry2* (decrease) transcripts were observed only in comparisons using specifics ZTs of light phases (Figs 7A and 8A). Intriguingly, Per1 transcripts levels were increased or decreased in hippocampus of epileptic rats depending on whether they were compared with specific ZT points of light and dark phases, respectively (Fig 9A).

In relation to ZT12 epileptic rats, we also observed that the different comparisons with naive group had effects on experimental results for Bmal1, Per1, Per3, Cry1 and Cry2 (Figs 4B–9B) but not for Clock. In fact, Clock transcripts were also significantly decreased in the hippocampus of epileptic rats in all comparisons to the naive group (Fig 4B). Per3 was increased in ZT12 only when compared with naive rats correspondent to ZT points of the dark phase (Fig 5B). Bmal1 was decreased when compared with all ZT of naive group, with exception for ZT12 and ZT16 (Fig 6B). Significant increases in Cry1 transcripts were observed only in comparisons using specifics ZTs of light phases (Fig 7B), while the CRY2 decreased was significant in comparisons using ZT of light phases and specifics ZT of dark phase (Fig 8B). Per1 transcripts were significantly increased only in comparisons with ZT of light phases (Fig 9B).

## Discussion

Studies that aim to measure transcript levels, particularly using RT-qPCR, must use proper reference genes to avoid erroneous conclusions [5,38,40]. To the best of our knowledge, this is the first study that investigated, systematically, suitable reference genes for circadian analysis in hippocampus of Wistar rats. The 8 candidate reference genes were ranked for expression stability in the hippocampus of rats sacrificed at six different ZT of a 12:12 light-dark cycle. The slight differences in geNorm and NormFinder ranking probably were due to specific mathematical algorithms used by the software. The pairwise variation value (V2/3) below of cutoff (0.15) indicated that normalization of target genes with a combination of the two best genes is sufficient. This is especially important considering that the most of circadian studies perform normalization using a single reference gene [28,32,41–43]. Thus, the best combination of reference genes indicated was *Tubb2a*/*Rplp1* and *Tubb2a*/*Ppia* by geNorm and NormFinder softwares, respectively. In fact, when we used these combinations of genes separately to normalize the *Per1* transcripts amounts in hippocampus of rats at different ZT, we observed a similar diurnal oscillation pattern of gene expression (Fig 2). In the literature, we found reported reliable reference genes for circadian studies in retina, adrenal glands, liver and leukocytes [44–46]. However, since reference genes suitable for one tissue or species/strain should not automatically be used for normalization in another, our data are unique and provide a useful reference for researchers that search for stable reference genes in hippocampus of Wistar rats.

Following, we used *Tubb2a*/*Rplp1* as normalizers to analyze the temporal variation of core clock transcript levels in the hippocampus of rats sacrificed at different times thoroughly a 24h period. Supporting previous reports, we observed that *Per1*, *Per3*, *Cry1*, *Cry2* and *Bmal1* are expressed in an oscillating manner in this structure (Fig 3). Anti-phasic patterns of *Bmal1* compared with *Per1*, *Per3* and *Cry1* are consistently observed in the SCN [47–49], which can be explained by the actions of these genes products in transcriptional/translational feedback loops of core clock mechanism. However, Jilg et al. [27] observed that mRNAs of clock genes *Cry1*, *Cry2* and *Bmal1* (but not of *Per1*) are simultaneously elevated in the hippocampus during late night, suggesting a common mechanism of transcriptional regulation of these genes in hippocampus. Regardless the *Clock* expression, we were unable to show significant alterations over a 24h period (Fig 3). Although a non-rhythmic *Clock* expression had already been reported in the SCN and some peripheral oscillators [42], our findings contrast with previous studies showing diurnal rhythms for *Clock* expression in hippocampus of rats [27,32,42,50]. This divergent result could be explained by differences in experimental design, including species/strain, photoperiod or statistical software for circadian analysis.

Although we still do not know much about the functional significance of the rhythm’s clock genes expression in hippocampus, it is probably associated to a local temporal program that control morphological and physiological circadian changes in this structure. In fact, hippocampal diurnal rhythms are observed in dendritic patterning and spine density of neuron [51,52], neurogenesis [53] and long-term potentiation [50], which must be implicated in the rhythm of hippocampal-dependent learning, memory formation and aging [54–59].

### Effects of diurnal variation on differential gene expression analysis in hippocampus of epileptic rats

The assessment of stochastic variation of transcript abundance by analyzing multiple samples from the same tissue/individual highlights a number of potential biological and technical factors that interfere in gene expression [10,60]. These studies revealed that the gene pathways and functions most closely associated with high baseline variance reflects functions that are sensitive to environmental cues, such as circadian rhythm [61]. However, the impact of these variations on differential gene expression studies in general and, particularly, in epilepsy has been underscored. Here, we verified that diurnal expression is an important source of variability on differential gene expression analysis in hippocampus of epileptic rats. In fact, we show that differences at ZT could have effects on experimental results for *Bmal1*, *Per1*, *Per3*, *Cry1* and *Cry2* (Figs 5–9) but not for *Clock* (Fig 4). Indeed, only the clock gene did not confirm a diurnal pattern of expression in hippocampus of naive rats. This is of great relevance considering that the components of the core clock mechanism regulate circadian rhythms in genome-wide mRNA expression. In fact, although there is no estimate for the fraction of the hippocampal transcriptome that shows circadian variation, a total of 2,927 genes were identified as circadian regulated in mouse prefrontal cortex [62]. Thus, it may be hypothesized that part of the controversies in differential expression studies of epileptogenic process is due to differences in the time of sampling. Interesting, some of the genes with divergent expression results for epileptogenic process, such as *Il-1β* [63–65], *Npy* [66,67] and *Cox-2* [68,69], have been reported presenting a rhythmic expression in different organs [70–72]. Our data highlight the importance of avoiding genes with circadian expression which are out of phase when comparing experimental and control groups. Moreover, since the circadian phase difference may also affect other processes underlying epileptogenesis, such as neurogenesis, inflammation and neurodegeneration [53,73], we strongly recommend to take the time of day in consideration when designing all types of experiments.

Regarding the differential expression of core clock genes, we observed that only the *Clock* transcript abundance presented a consistent alteration (decrease) in epileptic rats (ZT8 or ZT12) for all comparisons with the naive group, suggesting that a subexpression of *Clock* may be involved in epileptogenicity, possibly due to its role in gene expression regulation [74,75]. *Clock/Clock* mutants display in midbrain ventral tegmental area a decreased expression of the 1 subunit of the GABAA receptor and voltage-gated potassium channel (*KCNQ2*); and have increased levels of the *GLUR1* subunit of the AMPA glutamate receptor, which could contribute to the observed increase in neuronal excitability of epileptic brains [75]. Interestingly, the same pattern of these gene expression alterations is seen in the hippocampus of different models of epilepsy, and may enhance epileptogenicity [76–80].

The interpretation of functional significance of *Bmal1*, *Per1*, *Per3*, *Cry1* and *Cry2* altered expression here observed in epileptic rats is jeopardized because the results change depending on the ZT of both naive and epileptic rats in the comparison. To the best of our knowledge, two reported studies investigated whether the core clock genes expression (*Bmal1* and *Per1*, only) is modulated in epilepsy. Gerstner el al. [81] showed that seizure activity itself does not influence hippocampal *BMAL1* expression, despite of *BMAL1* gene regulates baseline threshold for seizures. Eun et al. [82] observed that *Per1* expression is induced in hippocampus of rats submitted to two different seizure induction protocols. In agreement, we also observed that *Per1* expression is increased in epileptic rats (ZT8 and ZT12) while the *Bmal1* is unchanged in hippocampus of epileptic rats (ZT8, only) when compared with naive rats correspondent to ZT points of the light phase. However, future studies examining diurnal profile gene expression in epilepsy coupled to functional assays will be important to disentangle the involvement of clock genes on generation or maintenance of seizures.

In conclusion, our study provides relevant information for experimental design and interpretation involving differential expression studies in hippocampus of epileptic rats. The investigators should keep all animals rigorously under the same environmental synchronizers and collect samples of experimental and control groups in a consistent and narrow time frame. In situations where the strict application of a narrower time for sample collection is not possible, such as for large experiments that require a lengthy sample collection times and replicates, we strongly recommend to include a previous step in the experimental design to evaluate if the target gene present a temporal cycle expression in such tissue/cell. In addition, we identified suitability reference genes for circadian studies in hippocampus of rats and suggested that a subexpression of *Clock* may be involved in epileptogenicity.
